# Diet of dingoes and other wild dogs in peri-urban areas of north-eastern Australia

**DOI:** 10.1038/srep23028

**Published:** 2016-03-11

**Authors:** Benjamin L. Allen, Erin Carmelito, Matt Amos, Mark S. Goullet, Lee R. Allen, James Speed, Matt Gentle, Luke K.-P. Leung

**Affiliations:** 1 Biosecurity Queensland, Queensland Department of Agriculture and Fisheries, Toowoomba, Queensland 4350, Australia; 2 The University of Southern Queensland, Institute for Agriculture and Environment, Toowoomba, Queensland 4350, Australia; 3 The University of Queensland, School of Agriculture and Food Sciences, Gatton, Queensland 4343, Australia; 4 FeralsOut, Kippa-Ring, Queensland 4021, Australia

## Abstract

Knowledge of the resource requirements of urban predators can improve our understanding of their ecology and assist town planners and wildlife management agencies in developing management approaches that alleviate human-wildlife conflicts. Here we examine food and dietary items identified in scats of dingoes in peri-urban areas of north-eastern Australia to better understand their resource requirements and the potential for dingoes to threaten locally fragmented populations of native fauna. Our primary aim was to determine what peri-urban dingoes eat, and whether or not this differs between regions. We identified over 40 different food items in dingo scats, almost all of which were mammals. Individual species commonly observed in dingo scats included agile wallabies, northern brown bandicoots and swamp wallabies. Birds were relatively common in some areas but not others, as were invertebrates. Dingoes were identified as a significant potential threat to fragmented populations of koalas. Dietary overlap was typically very high or near-identical between regions, indicating that peri-urban dingoes ate the same types or sizes of prey in different areas. Future studies should seek to quantify actual and perceived impacts of, and human attitudes towards, peri-urban dingoes, and to develop management strategies with a greater chance of reducing human-wildlife conflicts.

The encroachment of urbanisation into rural or wilderness areas has led to increased human-wildlife conflicts around the world^1^,^2^. Many wildlife species must now adapt to peri-urban ecosystems or be excluded from them. Large predators, such as bears (*Ursus arctos*) and wolves (*Canis lupus*), typically experience greater difficulty adapting to peri-urban ecosystems given their large home range and specialist prey requirements, which are often not met in peri-urban ecosystems^3^,^4^. However, smaller predators with more flexible resource requirements and generalist diets are becoming increasingly common in urban areas^4^,^5^,^6^,^7^. Understanding the food and prey resources utilised by these predators can improve our understanding of their ecology and assist town planners and wildlife management agencies in developing approaches that alleviate human-wildlife conflicts^8^.

Dingoes (*Canis lupus* ssp. *dingo*) were the largest terrestrial predator on mainland Australia when Europeans colonised the continent in the late 1700s^9^. Dingoes have since interbred with domestic dogs (*Canis familiaris*) subsequently brought to Australia, and ‘wild dogs’ (i.e. pure dingoes and/or dingo-dog crosses) are now very common^10^. Wild dogs are presently distributed across about 85% of Australia^11^, and inhabit almost all peri-urban areas within this extended range^12^. Human conflict with wild dogs in peri-urban areas typically relates to wild dog predation of small livestock and poultry, fouling and underuse of recreational amenities, and concern for zoonoses and wild dog attacks on people^13^,^14^. Knowledge of wild dog diet and prey preferences in rural or wilderness areas suggests that peri-urban wild dogs might also be serious predators of small to medium-sized native mammals^15^,^16^, which may also be threatened by habitat fragmentation and other processes affecting peri-urban ecosystems^17^,^18^,^19^,^20^. Despite the great potential for economic, environmental and social impacts of wild dogs in peri-urban areas of Australia^21^, little empirical information exists on their ecology or management and empirical research on peri-urban wild dogs is in its infancy^12^.

In this study, we examine the remains of food and dietary items identified in scats of wild dogs in peri-urban areas of north-eastern Australia to better understand their resource requirements and the potential for wild dogs to threaten locally fragmented populations of native fauna. Our primary aim was to determine what peri-urban wild dogs eat, and whether or not this differs between regions. We expected wild dogs to eat a wide variety of food items inclusive of mammals, reptiles and birds. Given their propensity to exploit human-sourced foods^22^,^23^, we also expected anthropogenic foods to occur commonly in wild dog diets. Knowledge of wild dog diets may also be useful for identifying native prey species that may be threatened by wild dog predation and for determining whether human-sourced food contributes to sustaining wild dog populations.

## Results

We collected a total of 277 peri-urban wild dog scats from north Queensland (NQ, with 229 from Mt Stuart Training Area, MSTA) and another 269 from peri-urban areas of south-east Queensland (SEQ). An additional 85 scats were collected from nearby rural areas in the Brisbane Valley (BV) to enable comparison of urban and rural diets within the SEQ region. Mammals (of all sizes) were recorded much more frequently than other food or prey items (Table 1). Individual species commonly observed in wild dog scats included agile wallabies (*Macropus agilis*), northern brown bandicoots (*Isoodon macrourus*) and swamp wallabies (*Wallabia bicolor*). Birds were relatively common in some areas (SEQ = 20% occurrence) but not others (<4% in all other areas), as were invertebrates (range = 0–9% occurrence). The most commonly occurring prey size class in wild dog diets from NQ were large mammals >10,000 g mean adult body weight (Table 2), such as agile wallabies (54% occurrence), and medium-sized mammals (2,000–10,000 g), such as bandicoots (30% occurrence). Wild dogs in SEQ had a more diverse diet, with relatively similar amounts of mammalian prey from different size classes (Table 2). Medium-sized mammals were most common in BV (58% occurrence).

Assessment of dietary overlap between wild dog populations inhabiting different areas showed highly variable results when comparing individual mammal species (Pianka’s index *O* = 0.07–0.95), as expected (Table 3). However, when mammalian size classes were compared, dietary overlap between populations was consistently much higher (*O* = 0.52–1.00). Unsurprisingly, comparisons between NQ and MSTA13 indicated complete overlap given that data from MSTA13 represents an 83% sub-sample of our NQ data. Comparison of samples collected in MSTA in 2013 (MSTA13) with those collected a decade earlier in MSTA in 2002^24^ (MSTA02) showed an almost identical degree of overlap (*O* = 0.94). Dietary overlap was also very high between SEQ and NQ regions (*O* = 0.92). The least degree of overlap was observed between wild dog scat samples collected in MSTA02 and BV (*O* = 0.52, Table 3).

## Discussion

Our results demonstrate that peri-urban dingoes and other wild dogs eat a wide variety of prey items, from invertebrates to cattle (*Bos taurus* and *B. indicus*; Tables 1 and 2). Most prey items were terrestrial species, although arboreal and fossorial species also occurred incidentally. Small to medium-sized mammals occurred most commonly, birds were common only in SEQ, and reptiles were seldom detected in wild dog scats (Table 1). In general, wallabies (*Macropus agilis*, *Wallabia bicolor*), bandicoots (*Isoodon macrourus*) and rats (*Rattus* spp.) appeared to be the most important prey items for wild dogs in peri-urban areas. Dietary overlap was typically very high between regions (Table 3, Fig. 2), indicating that peri-urban wild dogs ate the same types or sizes of prey in different regions.

**Figure 1:**
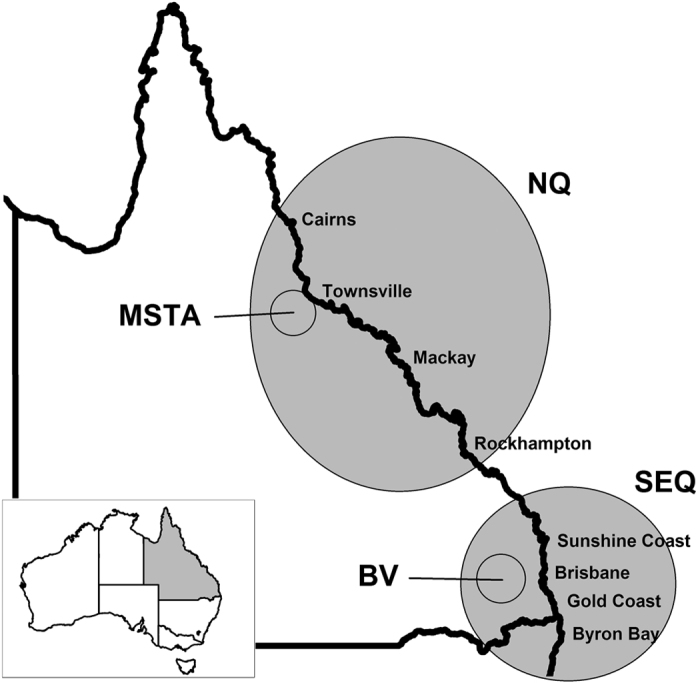
Location of study sites (shaded areas) and major cities on north-eastern Australia. Map created new in ArcGIS v10.1 (ESRI Inc.).

**Figure 2:**
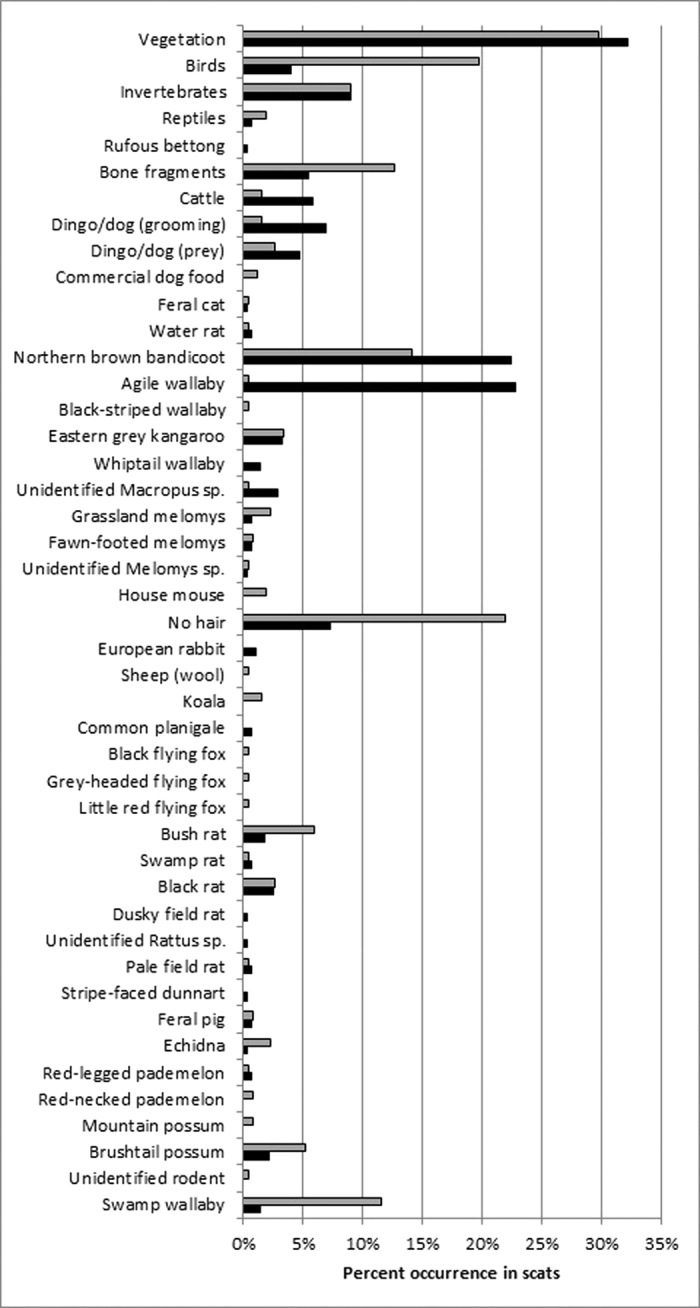
Comparison of food items (% occurrence) found in wild dog scats from north Queensland (NQ, black bars) and south-east Queensland (SEQ, grey bars), 2013–2015.

Some bandicoot and wallaby species are very common and widely distributed throughout eastern Australia^25^, and have previously been identified as primary prey for wild dogs in rural and wilderness areas^24^,^26^,^27^,^28^,^29^,^30^. Our results confirm that these species are not only present in peri-urban areas, but are also frequently consumed by wild dogs there (Table 1, Fig. 1), suggesting that rural and peri-urban wild dog populations may share similar prey preferences. However, overlap in wild dog diet between BV (a non-urban wilderness area near SEQ) and SEQ was lower than most other areas (Table 3), suggesting that although still quite high (*O* = 0.82), dietary overlap may be more consistent between peri-urban areas in different regions than between rural and peri-urban areas in the same region. Mammal assemblages differ between north and south Queensland^25^, although species of similar size and shape are present in both areas, which is why wild dog predation of species grouped into body size classes were similarly high between regions (Table 3, Fig. 2). Available data on wild dog diet in Australia have increased substantially over the last 15 years^15^, though there has been no systematic nationwide comparison of wild dog diets from different areas, as there has been for some specific regions^27^.

Koalas (*Phascolarctos cinereus*) and pademelons (*Thylogale* spp.) were among the threatened species found in peri-urban wild dog scats. Although a low occurrence of a given species in wild dog scats might imply that wild dogs are not a threat to them, further information (beyond scat data) is required before such results should be dismissed as inconsequential^15^. Detecting a prey species in wild dog scats would not be expected if wild dogs had already contributed to their local extinction, and prey absence in scats might also simply mean that alternative species more preferred by wild dogs (such as macropods or bandicoots) were available and preferentially eaten at the time of scat sampling (Table 2). Moreover, greater variety of extant mammals probably dampens the effects of wild dog predation^31^,^32^,^33^ on a given species because of greater opportunity to switch between prey as one species or another becomes unavailable^34^. But because peri-urban wild dogs appear largely reliant on just one or two primary prey species in any given location (Tables 1 and 2), the risk of hyperpredation^35^ on fragmented populations of koalas and other locally threatened fauna may still be severe.

Data from this study and associated koala research suggest that peri-urban wild dogs have the capacity to seriously threaten local populations of koalas under certain conditions^36^,^37^, as is the case in the Hays Inlet area of Brisbane in SEQ^38^. To illustrate this, if one scat represents the prey eaten by an individual wild dog in the previous 24 hours, then 1% occurrence (Table 1) theoretically represents at least 1 koala killed every 100 days per wild dog per year, or 4 koalas per wild dog per year. Given that approximately 15 wild dogs (or 2–3 packs) are present in the ~10 km^2^ area of available bushland (B. Allen, unpublished data), 1% occurrence in scats could represent predation of 55 individual koalas within the Hays Inlet area each year. Approximately 200 koalas are present in this isolated fragment of bushland at any one time and wild dog predation has been the cause of death for at least 106 of 232 (45.7%) koalas found dead in the area between March 2013 and April 2015^38^. Given the slow reproductive rate of koalas and the other factors limiting their local abundance (i.e. disease and habitat availability^38^), these data support previous findings and suggest that even relatively minor levels of wild dog predation can threaten fragmented populations of native fauna with local extinction in peri-urban areas^36^. A greater understanding of predator-prey interactions in peri-urban bushland fragments is required to understand the effects of wild dogs on iconic native species of high conservation value.

Our expectation that wild dogs would consume relatively large amounts of human-sourced foods, such as rubbish or pet food, was not supported by the data (Table 1, Fig. 2). This may be because wild dogs do not consume these foods in substantial quantities. Alternatively, these food items might be more completely digestible and unlikely to be detected using the methods with which our samples were assessed (see below). Our sampling approach, which sought to avoid scats that appeared to originate from domestic dogs, may have also excluded wild dog scats that looked like domestic dogs scats. Peri-urban wild dogs are known to travel ‘house to house’ at night^12^, with anecdotal reports of wild dogs consuming pet food left outside for pets and raiding rubbish bins. Attempting to separately identify scats of wild dogs from those of domestic dogs in peri-urban areas is difficult, if not impossible, given that they utilize the same available landscapes, are closely related, may consume similar food items, and deposit scats of similar appearance in the same places. Future studies attempting to do this might focus efforts on observing wild dog feeding behaviour, quantifying wild dog visitation rates at domestic dog feeding locations, or assessing food and prey items present in the stomachs of known-origin dogs (wild or domestic) trapped or otherwise sourced.

In conclusion, our data have shown that wild dogs eat a wide variety of predominately mammalian prey in peri-urban areas, and may potentially threaten fragmented populations of some local native fauna in these areas. These findings increase our understanding of wild dog ecology in peri-urban areas and have important management implications. Limiting access to food subsidies is a common way of reducing human-wildlife conflicts^23^,^39^. However, peri-urban wild dogs do not appear to be reliant on human-sourced foods, which means that reducing or eliminating access to these food sources is unlikely to influence wild dogs to any substantial degree. The impacts of dingoes and other wild dogs on threatened fauna are also underappreciated^15^,^37^,^40^,^41^, or at least understudied, especially in peri-urban areas where domestic dog impacts are most often studied or inferred^36^,^42^. Future studies should seek to better quantify actual and perceived impacts of, and attitudes towards, peri-urban dingoes and other wild dogs, and management strategies with a greater chance of reducing human-wildlife conflicts.

## Methods

### Ethics statement

The dingo is considered native wildlife under the *Nature Conservation Act 1992*, and is protected in national parks. Elsewhere in Queensland dingoes and other wild dogs are declared pest species under the *Land Protection (Pest and Stock Route Management) Act 2002*. Approval to undertake the project was granted by the Department of Agriculture, Forestry and Fisheries Animal Ethics Committee (AEC permit number: CA 2013/04/685), and the project was conducted in accordance with this approval. Most areas sampled were freely accessible to the public, and ‘permission to enter’ was not necessary. However, where access was restricted (e.g. MSTA), we first obtained permission from the landowner or approving officer prior to collecting scats.

### Study sites

Scats were collected in several locations across Queensland, Australia, including peri-urban areas of Atherton, Cairns, Townsville, Mackay and Rockhampton (pooled together as ‘north Queensland’, NQ), and also the Sunshine Coast, north Brisbane, the Gold Coast and the Tweed Coast (pooled together as south-east Queensland, SEQ; Fig. 1). Previous studies have indicated that approximately 30 scats are needed to reliably describe wild dog diets^43^,^44^. Hence, we considered sample sizes from each individual area too small for meaningful analyses and instead grouped them together into two regions representing similar habitat types and mammal assemblages. Most scats from the Townsville area were collected at the Mt Stuart Training Area (MSTA; a restricted-access military reserve), and sub-samples from this area were analysed separately in some cases to aid comparison with previously published information from MSTA^24^. Scats collected in rural or bushland areas of the Brisbane Valley (BV), about 50 km west of Brisbane, were excluded from the SEQ group because they were not collected in peri-urban areas. Thus, we analysed scat samples from four geographic locations within two regions, and scats were sampled in two periods (2002 and 2013) from one of these locations (MSTA).

### Scat collection

Scats were collected between September 2012 and June 2015 (most in 2013 and 2014) from a wide variety of road reserves, bushland fragments, beach dunes, riparian areas, manicured parklands and other areas where wild dogs were known or expected to occur in peri-urban areas^12^. In other words, we primarily sought scats from bushland patches in or near the edge of built-up residential and industrial areas known to harbour wild dogs. Scats were placed in a brown paper bag, and the date and location (latitude, longitude) of the scat was recorded. Scats were then air-dried for several weeks before being sent to a professional service provider who sterilized, then washed each scat to extract hair, bone and other material suitable for prey identification. Mammalian food items were identified to the lowest taxonomic level possible according to diagnostic characteristics of mammalian hair^45^,^46^. Dingoes or wild dogs in our study sites share peri-urban areas with domestic or pet dogs and introduced red foxes (*Vulpes vulpes*)^12^,^47^, and the scats of all these canids can appear very similar^48^. Dog scats were differentiated from fox scats based on their size, appearance, odour and placement. We excluded domestic dog scats primarily by location and appearance (e.g. scats at a person’s letterbox containing nothing but the remains of processed dog food). However, we cannot discount the possibility that some of our scat samples come from domestic dogs, especially where these dogs are free-roaming and consume fauna. Nor can we discount the possibility that some wild dog scats were incorrectly excluded as domestic dog scats, especially where wild dogs were frequenting built-up areas and consuming processed dog food intended for domestic dogs.

### Data analyses

Prey or food items present in dog scats were expressed as the percent occurrence in scats, which is best suited to describe diets where most items occur relatively infrequently^49^. Mammalian prey items were grouped into body weight categories of 0–100 g, 100–500 g, 500–2,000 g, 2,000–10,000 g and >10,000 g, based on average adult prey weights^25^. Category bandwidths were arbitrarily chosen to reflect the broad prey preferences of wild dogs^50^, however, results are unlikely to be influenced by the choice of alternative size categories^15^. We compared dietary overlap between populations of wild dogs using Pianka’s Index of overlap^51^, which is commonly used to compare diets between different predators in the same area and/or populations of the same predator in different areas^43^,^52^,^53^. Index values of 0 represent completely different diets, and index values of 1 represent identical diets or complete overlap. Because vertebrates (and especially mammals) occurred most frequently (Tables 1 and 2), we first compared dietary overlap using data from all vertebrate prey items (e.g. mammals, reptiles and birds). However, mammal assemblages differ substantially between geographic regions^25^ and the identity of bird and reptile species was often unavailable. Thus, we further compared mammalian prey size classes across regions to investigate whether or not wild dogs were consuming the same type (or size) of mammalian prey, regardless of whether or not a particular taxon was present in each region. Human-sourced foods are defined as things like pet food, rubbish, processed meat, bread etc, and do not include domesticated livestock such as poultry, sheep or cattle. Analyses and inferences were constrained to the simple and robust approaches described above given the scat sampling procedures used and our modest sample sizes.

## Additional Information

**How to cite this article**: Allen, B. L. *et al.* Diet of dingoes and other wild dogs in peri-urban areas of north-eastern Australia. *Sci. Rep.* **6**, 23028; doi: 10.1038/srep23028 (2016).

## Figures and Tables

**Table 1:** The proportion of different food items detected in wild dogs scats from north Queensland (NQ, N = 277), south-east Queensland (SEQ, N = 269), Mount Stuart Training Area in 2013 (MSTA13, N = 229; a sub-sample of those from NQ), Mount Stuart Training Area in 2002 (MSTA02, N = 118) and the Brisbane Valley (BV, N = 85).

Type	Common name	Taxonomic name	Size class	NQ	SEQ	MSTA13	MSTA02	BV
Mammals	Common planigale	Planigale maculata	1	0.01	0.00	0.00	0.00	0.00
	Eastern chestnut mouse	Pseudomys gracilicaudatus	1	0.00	0.00	0.00	0.00	0.06
	Fawn-footed melomys	Melomys cervinipes	1	0.01	0.01	0.01	0.00	0.00
	Grassland melomys	Melomys burtoni	1	0.01	0.02	0.01	0.00	0.00
	House mouse	Mus musculus	1	0.00	0.02	0.00	0.00	0.02
	Stripe-faced dunnart	Sminthopsis macroura	1	0.00	0.00	0.00	0.00	0.00
	Unidentified Melomys sp.	Melomys sp.	1	0.00	0.00	0.00	0.00	0.00
	Unidentified rodent/dasyurid	Unidentified rodent	1	0.00	0.00	0.00	0.00	0.00
	Black flying fox	Pteropus alecto	2	0.00	0.00	0.00	0.00	0.00
	Black rat	Rattus rattus	2	0.03	0.03	0.03	0.00	0.00
	Bush rat	Rattus fuscipes	2	0.02	0.06	0.02	0.00	0.05
	Dusky field rat	Rattus sordidus	2	0.00	0.00	0.00	0.00	0.00
	Grey-headed flying fox	Pteropus poliocephalus	2	0.00	0.00	0.00	0.00	0.00
	Little red flying fox	Pteropus scapulatus	2	0.00	0.00	0.00	0.00	0.00
	Pale field rat	Rattus tunneyi	2	0.01	0.00	0.01	0.00	0.00
	Sugar glider	Petaurus breviceps	2	0.00	0.00	0.00	0.00	0.00
	Swamp rat	Rattus lutreolus	2	0.01	0.00	0.00	0.00	0.01
	Unidentified Rattus sp.	Rattus sp.	2	0.00	0.00	0.00	0.00	0.01
	Water rat	Hydromys chrysogaster	2	0.01	0.00	0.01	0.00	0.00
	Echidna	Tachyglossus aculeatus	3	0.00	0.02	0.00	0.00	0.00
	European rabbit	Oryctolagus cuniculus	3	0.01	0.00	0.01	0.06	0.00
	Mountain possum	Trichosurus caninus	3	0.00	0.01	0.00	0.00	0.00
	Northern brown bandicoot	Isoodon macrourus	3	0.22	0.14	0.21	0.04	0.58
	Brushtail possum	Trichosurus vulpecula	4	0.02	0.05	0.00	0.04	0.04
	Feral cat	Felis catus	4	0.00	0.00	0.00	0.02	0.00
	Koala	Phascolarctos cinereus	4	0.00	0.01	0.00	0.00	0.00
	Red-legged pademelon	Thylogale stigmatica	4	0.01	0.00	0.01	0.00	0.00
	Red-necked pademelon	Thylogale thetis	4	0.00	0.01	0.00	0.00	0.00
	Rufous bettong	Aepyprymnus rufescens	4	0.00	0.00	0.00	0.00	0.00
	Agile wallaby	Macropus agilis	5	0.23	0.00	0.25	0.57	0.00
	Allied rock-wallaby	Pertrogale assimilis	5	0.00	0.00	0.00	0.04	0.00
	Black-striped wallaby	Macropus dorsalis	5	0.00	0.00	0.00	0.00	0.00
	Cattle	Bos taurus	5	0.06	0.01	0.06	0.02	0.00
	Dingo/dog (prey)	Canis sp. (prey)	5	0.05	0.03	0.06	0.01	0.00
	Dingo/dog (grooming)	Canis sp. (grooming)	N/A	0.07	0.01	0.04	0.00	0.00
	Eastern grey kangaroo	Macropus giganteus	5	0.03	0.03	0.04	0.02	0.07
	Feral goat	Capra hircus	5	0.00	0.00	0.00	0.02	0.00
	Feral pig	Sus scrofa	5	0.01	0.01	0.01	0.01	0.00
	Swamp wallaby	Wallabia bicolor	5	0.01	0.12	0.02	0.00	0.02
	Unidentified deer		5	0.00	0.00	0.00	0.00	0.18
	Unidentified Macropus sp.	Macropus sp.	5	0.03	0.00	0.03	0.04	0.00
	Whiptail wallaby	Macropus parryi	5	0.01	0.00	0.02	0.15	0.00
Other	Birds		N/A	0.04	0.20	0.00	0.03	0.00
	Reptiles		N/A	0.01	0.02	0.00	0.00	0.00
	Invertebrates		N/A	0.09	0.09	0.00	0.02	0.01
	Bone fragments		N/A	0.05	0.13	0.06	0.00	0.01

**Table 2:** Proportion of mammals, categorised by size class, found in wild dog scats from north-eastern Australia (see Table 1 for explanation of site abbreviations).

Mammal size class (g)	NQ	SEQ	MSTA13	MSTA02	BV
1 (0–100)	0.04	0.09	0.03	0.00	0.08
2 (100–500)	0.08	0.15	0.07	0.00	0.07
3 (500–2,000)	0.30	0.25	0.23	0.08	0.58
4 (2,000–10,000)	0.05	0.18	0.02	0.05	0.04
5 (>10,000)	0.53	0.33	0.49	0.75	0.27

**Table 3:** Pianka’s index values for comparisons of the proportion of individual vertebrate prey items (left) and mammalian size classes (right) found in wild dog scats from north-eastern Australia.

	NQ	SEQ	MSTA13	MSTA02	BV
NQ					
SEQ	0.55|0.92				
MSTA13	0.95|1.00	0.41|0.90			
MSTA02	0.69|0.91	0.10|0.76	0.75|0.94		
BV	0.61|0.82	0.47|0.82	0.69|0.77	0.07|0.52	
